# Differences in athletic identity, sport participation, and psychosocial factors following anterior cruciate ligament rehabilitation in youth athletes

**DOI:** 10.3389/fpsyg.2023.1303887

**Published:** 2024-01-08

**Authors:** James McGinley, Emily Stapleton, Emily Gale, Hannah Worrall, Caroline Podvin, Henry B. Ellis, Philip L. Wilson, Sophia Ulman

**Affiliations:** ^1^Center for Excellence in Sports Medicine, Scottish Rite for Children Orthopedic and Sports Medicine Center, Frisco, TX, United States; ^2^Department of Psychology, Scottish Rite for Children, Frisco, TX, United States; ^3^Department of Orthopaedic Surgery, University of Texas Southwestern Medical Center, Dallas, TX, United States

**Keywords:** athletic identity, youth, sport psychology, rehabilitation, anterior cruciate ligament, return-to-sport

## Abstract

**Introduction:**

While youth sports benefits the developing athlete, athletes may also be subject to injury and subsequent return-to-sport protocols. The current return-to-sport criteria emphasize physical measures; however, psychological measures may also be valuable to inform providers of an athlete’s readiness. One such measure is athletic identity defined as the degree to which an individual identifies with the athlete role. To better understand athletic identity in return-to-sport, this study aimed to identify relationships and trends between the Athletic Identity Measurement Scale (AIMS), demographic variables, sport participation measures, and the Athletic Coping Skills Inventory-28 (ACSI-28) in youth athletes during rehabilitation following anterior cruciate ligament reconstruction (ACLR).

**Methods:**

A retrospective review was completed of patients who underwent ACLR at a sports medicine clinic between October 2019 and May 2021. Patients responded to a series of patient reported outcomes (PROs) regarding physical and psychological function at a pre-surgical baseline and after 1 year of rehabilitation. Patients were then divided into groups of high/low AIMS and an increased/decreased AIMS between 1 year and baseline for comparison. Independent samples t-tests and ANOVAs were performed as appropriate with a 95% confidence interval.

**Results:**

In the final sample, 87 patients (15.3 ± 1.8 years) were included, with 51.7% being females. Total AIMS scores decreased from 50.3 to 47.5 over rehabilitation (*p* = 0.019). Furthermore, results indicated that nearly all AIMS scores decreased during rehabilitation, with none showing an increase; however, not all domains were significant. Conversely, all sport participation and coping ability PROs increased over time points except for ACSI-Confidence and Achievement Motivation. Generally, those in the groups with high AIMS and an increase in AIMS also had higher scores in physical function and coping ability PROs, with the groups separated by high/low AIMS exhibiting more frequent statistical significance.

**Discussion:**

Given these results, it appears that athletes may lose identification with the athlete role after ACLR and struggle even 1 year for rehabilitation, but those who recover athletic identity the best may also be those able to cope most effectively with the stressors induced by injury.

## 1 Introduction

Youth sport offers myriad benefits early in life by developing physical health, teamwork skills, and a robust network of peer relationships. Sport may also supplement psychological health by reducing symptoms of depression, anxiety, and stress (Eime et al., 2013; McKay et al., 2019; Panza et al., 2020). During the formative years of childhood and adolescence, sport and its associated social connections may comprise a prominent portion of a child’s personal identity. The extent of this phenomenon is characterized by Brewer et al. (1993) as athletic identity or the degree to which individuals identify with the athlete role.

To better gauge an athlete’s outcomes in return-to-sport, an understanding of a child’s dependence on and involvement in sport—their athletic identity—may be helpful. Athletes may be forced to alter or abandon their athletic identity during periods of situational change, such as injury and retirement from sport (Brewer and Cornelius, 2010; Newton et al., 2020). In fact, injury is a major cause of early retirement at all ages (Ardern et al., 2014; McKay et al., 2019). During periods of injury, the strong social support system that athletes build for themselves may falter as they abstain from training, often resulting in feelings of isolation and a loss of identity (Podlog et al., 2011; Von Rosen et al., 2018). These injured athletes may also develop depressive symptoms and fear as a result of their exclusion from sport and anticipation of their return (Park et al., 2023). While peers and others in their support system, such as athletic trainers, may benefit athletes’ psychological health through the rehabilitation process (DiSanti et al., 2018), negative feelings frequently persist. Importantly, returning to sport often produces both greater long-term physical function and happiness, highlighting the importance of returning as many athletes as possible to their pre-injury sport and competition level (Langford et al., 2009; Ardern et al., 2016; Hsu et al., 2021).

The current return-to-sport criteria primarily emphasize physical measures of readiness; however, psychological measures of rehabilitation, such as athletic identity, may also be valuable due to the influence of injury and rehabilitation on these metrics (Lochbaum et al., 2022). For example, depressive symptoms and loss of motivation over rehabilitation, which lead to worsened physical function, may be increased in those with high athletic identity (Christino et al., 2015; Morgan et al., 2020; Renton et al., 2021), particularly high athletic identity youth (Manuel et al., 2002; Chow et al., 2016; McKay et al., 2019; Edison et al., 2021; Nyland and Pyle, 2022). Athletes may also display greater catastrophizing thinking patterns and emotional trauma (Shuer and Dietrich, 1997; Padaki et al., 2018; Costa et al., 2020). Finally, coping ability is likely relevant to rehabilitation success; athletes who can cope more effectively may alleviate these depressive symptoms and trauma (Ardern et al., 2014; Samuel et al., 2015; Renton et al., 2021; Nyland and Pyle, 2022). Nevertheless, the relationship between coping ability and athletic identity is unclear as one study reported worsened coping in youth with high athletic identity (McGinley et al., 2022), while another study reported better coping skills in specialized athletes of all ages, which was associated with higher athletic identity (Christino et al., 2021). Overall, psychological measures of readiness appear to be related to one’s identity in sport, but expanded knowledge on specific positive and negative psychological factors is needed.

As physical function and psychological recovery from situational change appear to be related to athletic identity, it may be beneficial to gain an understanding of this characteristic over the course of rehabilitation (Edison et al., 2021; Lochbaum et al., 2022). However, the literature primarily provides data for athletic identity at pre-injury or immediately post-injury. As such, this study’s objectives were to determine (1) how athletic identity scores, sport participation, and coping ability change from pre-ACLR to 1-year post-ACLR, (2) how changes in athletic identity scores relate to sport participation and coping ability measures collected from pre-ACLR to 1-year post-ACLR, and (3) which measures are related to higher 1-year post-ACLR athletic identity scores.

## 2 Materials and methods

This study was conducted with approval from the University of Texas Southwestern Institutional Review Board (IRB; STU-2019-0701). As it was retrospective in nature, informed consent was not required and was waived.

### 2.1 Patients

A consecutive review was conducted on patients under 19 years who presented to a pediatric sports medicine practice and completed the standardized sports medicine intake and patient-reported outcome (PRO) measures between October 2019 and May 2021. Patients were included if they presented with an ACL injury and completed the Athletic Identity Measurement Scale (AIMS), a self-report measure used to quantify athletic identity (Brewer et al., 1993), both at pre-surgery and at 1-year post-ACL injury for the mid-to-late-rehabilitation analysis (Table 1).

**Table 1:** Patient demographics and sport characteristics.

Variable		*N* (%)
Sex	Female	45 (51.7)
	Male	42 (48.3)
Race	White	64 (73.6)
	Black or African American	17 (19.5)
	Non-White or Black	6 (6.9)
*Ethnicity*	Hispanic or Latino	24 (27.6)
	Non-Hispanic or Latino	63 (72.4)
*Competition Level*	School	38 (43.7)
	Select/Club	22 (25.3)
	Other	6 (6.9)
*Sport Participation*	Single-sport	52 (59.8)
	Multi-sport	23 (26.4)

*Non-White or Black may include Native American or Alaskan Native, Indian, Asian, or mixed race. The competition level was reported as the highest level achieved, with School as the lowest level and college as the highest level. Other competition levels may be described as recreational or non-competitive.

All surgical procedures were completed by two surgeons at a single sports medicine clinic. All patients were recommended for outpatient, supervised physical therapy after surgery. After 9 months of surgery, patients were initially reviewed and considered for clearance to the beginning stages of return-to-play.

### 2.2 Data acquisition

Demographics and sport participation measures were collected through a retrospective review at pre-ACLR and included age at presentation, sex, race, ethnicity, competition level (school, club/select, college, or other), and single- or multi-sport participation. Training volume (years in sport, days per week, weeks per year, and weeks off per year), recovery duration, and PROs were collected as standard-of-care at both pre-ACLR and 1-year post-ACLR from the patient’s intake form and/or surveys administered through the Outcome Based Electronic Research Database (OBERD; Universal Research Solution, LLC; Columbia, Missouri), an electronic PRO system validated in youth (Sabatino et al., 2019). PROs included the AIMS, the Hospital for Special Surgery Pediatric Functional Activity Brief Scale (Pedi-FABS), the Pediatric International Knee Documentation Committee Subjective Form (Pedi-IKDC), the Anterior Cruciate Ligament-Return to Sport after Injury scale (ACL-RSI), and the Athletic Coping Skills Inventory-28 (ACSI-28; McGinley et al., 2022). Patients were asked to respond to PROs related to function only in their current injured state at pre-ACLR.

### 2.3 Statistical analysis

The change in pre-operative and post-operative subscores and total scores was evaluated using paired samples t-tests. All athletes were then categorized into two groups based on the median AIMS score at 1-year post-ACLR. These groups were split by total AIMS scores greater than 50 (AIMS_H_) and less than 50 (AIMS_L_) similar to other relevant studies as no “high” or “low” AIMS cutoff score has been defined in the literature (Padaki et al., 2018; Costa et al., 2020; McGinley et al., 2022). Independent samples t-tests and ANOVAs were performed to compare demographics, sport participation, and PROs between the two groups. Then, athletes were divided into two new groups based on the change in AIMS scores (ΔAIMS) from pre-ACLR to 1-year post-ACLR. Those with any increase in AIMS scores (AIMS_INC_) were compared to those who had a decrease in AIMS scores (AIMS_DEC_). Independent samples t-tests and ANOVAs were performed as appropriate. A conventional 0.05 level of significance was set for all statistical tests.

## 3 Results

A total of 87 patients between the age of 10.8 and 18.9 (15.3 ± 1.8) years were analyzed at 1-year post-ACLR. As patients were only required to complete the AIMS for inclusion, no patients were excluded for other incomplete forms. Patients (N = 87) were reflective of the study region in race and ethnicity and primarily competed in a single sport (59.8%) at the school level (43.7%, Table 1). Only 12 out of 87 (14%) of patients were either referred to a psychologist for a consultation or provided an opportunity to speak with a psychologist in a clinic. Given this small percentage of patients, the effects of psychological intervention on the AIMS score were not evaluated.

The AIMS total score significantly differed by sex at pre-ACLR (*p = 0.034*); females had an average total AIMS score of 48.2 ± 9.5, while males had an average total AIMS score of 52.6 ± 9.9. However, 1-year post-ACLR (44.9 ± 13.2 females; 50.3 ± 12.6 males; *p = 0.054*), the decrease in AIMS total score between visits (−0.1 ± 0.2 females; 0.0 ± 0.2 males; *p = 0.541*) did not differ by sex. There were no significant differences in race or ethnicity groups for the total AIMS score at either timepoint or in the AIMS score change between time points. Similarly, no differences were found based on the competition level or sport participation status (single-sport vs. multi-sport participation).

When comparing individual AIMS items, at 1-year post-ACLR, all individual AIMS items decreased from pre-ACLR. However, only Question 7 (“Other people see me mainly as an athlete”), Question 10 (“I would be very depressed if I were injured and could not compete in sport”), and AIMS total scores exhibited a significant decrease (*p = 0.035, p = 0.005, p = 0.019;* Table 2). Average AIMS total scores were observed to be 50.3 ± 9.9 pre-ACLR and 47.5 ± 13.1 after 1 year of rehabilitation.

**Table 2:** AIMS total score and individual question ratings (mean ± SD) for all patients at pre-ACLR and 1-year post-ACLR.

**AIMS Question**	Pre-ACLR	Post-ACLR	*p* value
1. I consider myself an athlete.	6.7 ± 0.7	6.7 ± 1.0	0.911
2. I have many goals related to sport.	6.0 ± 1.4	5.9 ± 1.5	0.690
3. Most of my friends are athletes.	6.1 ± 1.5	5.9 ± 1.5	0.422
4. Sport is the most important part of my life.	5.2 ± 1.6	4.8 ± 1.9	0.080
5. I spend more time thinking about sport than anything else.	4.5 ± 1.8	4.2 ± 2.1	0.090
6. I need to participate in sport to feel good about myself.	3.9 ± 2.3	3.8 ± 2.3	0.673
7. Other people see me mainly as an athlete.	**5.6 ± 1.5**	**5.2 ± 2.0**	**0.035**
8. I feel bad about myself when I do poorly in sport.	4.4 ± 2.2	4.1 ± 2.3	0.195
9. Sport is the only important thing in my life.	2.8 ± 1.9	2.5 ± 1.8	0.187
10. I would be very depressed if I were injured and could not compete in sport.	**5.2 ± 2.1**	**4.4 ± 2.4**	**0.005**
**Total Score**	**50.3 ± 9.9**	**47.5 ± 13.1**	**0.019**

*Significance notated in bold. AIMS: Athletic Identity Measurement Scale. SD: Standard deviation. ACLR: Anterior cruciate ligament reconstruction.

Between pre-ACLR and post-ACLR, all PROs except ACSI-Confidence and Achievement Motivation increased; however, Pedi-FABS and ACSI-Concentration scores did not exhibit a significant increase though all other PROs were significant (Table 3).

**Table 3:** Change in outcome measures between time points.

	Pre-ACLR	Post-ACLR	
*Patient-Reported Outcome Measure*	*Mean ± SD*	*Mean ± SD*	*p value*
Pedi-FABS	21.4 ± 9.0	22.8 ± 6.9	0.321
Pedi-IKDC	**47.2 ± 19.6**	**91.4 ± 12.5**	**< 0.001**
ACL-RSI	**52.3 ± 26.2**	**77.5 ± 19.3**	**< 0.001**
ACSI-28	**58.2 ± 9.2**	**62.9 ± 11.9**	**< 0.001**
Coachability	**10.0 ± 2.0**	**10.6 ± 1.7**	**0.035**
Concentration	8.8 ± 1.9	9.0 ± 2.5	0.564
Confidence and Achievement Motivation	**9.9 ± 1.6**	**9.4 ± 2.1**	**0.044**
Coping	**7.4 ± 2.6**	**8.6 ± 2.5**	**0.001**
Freedom from Worry	**7.4 ± 3.1**	**8.4 ± 2.5**	**0.007**
Goal Setting and Mental Preparation	**6.9 ± 2.5**	**7.8 ± 3.0**	**0.004**
Peaking under Pressure	**7.7 ± 2.4**	**8.8 ± 2.6**	**0.001**

*Significance notated in bold. ACLR: Anterior cruciate ligament reconstruction. SD: Standard deviation. Pedi-FABS: Hospital for Special Surgery Pediatric Functional Activity Brief Scale. Pedi-IKDC: Pediatric International Knee Documentation Committee Subjective Form. ACL-RSI: Anterior Cruciate Ligament-Return to Sport after Injury scale. ACSI-28: Athletic Coping Skills Inventory-28.

As three patients did not change from baseline to 1-year time points and were too small of a sample for analysis, 84 patients were included and analyzed for change in AIMS scores, with 30 in the AIMS_INC_ group and 54 in the AIMS_DEC_ group. In this cohort, the number of weeks per year spent competing was significantly greater in the AIMS_INC_ group (*p = 0.016*; Table 4). ACSI-Peaking under Pressure was also greater in the AIMS_INC_ group (*p = 0.009*). Activity level, physical function, and ACSI scores other than Coachability were greater in the AIMS_INC_ group despite only Peaking under Pressure being significant (Table 4).

**Table 4:** Change in AIMS total score and outcome measures collected at 1-year post-ACLR.

	ΔAIMS		
Variable	AIMS_DEC_	AIMS_INC_	*p* value
*Demographics/Participation Volume*	*Mean ± SD*	*Mean ± SD*	
Age	15.4 ± 1.7	15.1 ± 1.9	0.495
Recovery Duration	272.0 ± 112.7	268.7 ± 77.6	0.902
Years Active in Primary Sport	6.7 ± 3.8	7.5 ± 3.5	0.371
Hours per Week	9.4 ± 5.9	8.5 ± 4.3	0.550
Days per Week	4.4 ± 1.6	4.6 ± 1.5	0.607
Weeks per Year	**27.6 ± 16.4**	**37.7 ± 14.1**	**0.016**
Weeks Off per Year	10.0 ± 10.3	9.3 ± 12.0	0.791
*PROs at 1-Year Post-ACLR*			
Pedi-FABS	21.9 ± 7.6	24.6 ± 4.8	0.182
Pedi-IKDC	88.9 ± 13.6	95.4 ± 8.7	0.072
ACL-RSI	75.0 ± 20.5	78.3 ± 18.0	0.570
ACSI-28	61.1 ± 12.4	65.4 ± 9.6	0.191
Coachability	10.6 ± 1.7	10.6 ± 1.7	0.965
Concentration	8.8 ± 2.6	9.4 ± 2.2	0.389
Confidence and Achievement Motivation	9.2 ± 2.2	9.5 ± 1.8	0.590
Coping	8.4 ± 2.6	8.8 ± 2.1	0.552
Freedom from Worry	8.1 ± 2.4	8.9 ± 2.6	0.222
Goal Setting and Mental Preparation	7.6 ± 3.0	8.3 ± 2.6	0.357
Peaking under Pressure	**8.2 ± 2.8**	**9.8 ± 1.6**	**0.009**

*Significant differences between AIMS groups are notated in bold. Groups split by increase (AIMS_INC_) or decrease (AIMS_DEC_) in total AIMS from baseline to 1 year. AIMS: Athletic Identity Measurement Scale. ACLR: Anterior cruciate ligament reconstruction. SD: Standard deviation. Pedi-FABS: Hospital for Special Surgery Pediatric Functional Activity Brief Scale. Pedi-IKDC: Pediatric International Knee Documentation Committee Subjective Form. ACL-RSI: Anterior Cruciate Ligament-Return to Sport after Injury scale. ACSI-28: Athletic Coping Skills Inventory-28.

Regarding high and low groups dichotomized by a total AIMS score of 50, there were 42 patients included in the AIMS_H_ group and 45 in the AIMS_L_ group. Groups did not show significant differences in any demographic or participation volume measures except for training days per week (*p = 0.033*) though the AIMS_H_ group consistently displayed a greater training volume. The AIMS_H_ group also showed greater Pedi-FABS (*p = 0.012*) and Pedi-IKDC (*p = 0.049*) scores at 1-year post-ACLR in addition to five ACSI measures, including ASCI-Total (*p = 0.028*; Table 5).

**Table 5:** High/low AIMS total score and outcome measures collected at 1-year post-ACLR.

	Post-ACLR		
Variable	AIMS_L_	AIMS_H_	*p* value
*Demographics / Participation Volume*	*Mean ± SD*	*Mean ± SD*	
Age	15.4 ± 1.7	15.2 ± 1.9	0.725
Recovery Duration	281.7 ± 118.9	256.9 ± 72.5	0.328
Years Active in Primary Sport	6.4 ± 3.8	7.8 ± 3.4	0.137
Hours per Week	8.9 ± 5.1	9.5 ± 5.7	0.630
Days per Week	**4.1 ± 1.6**	**4.9 ± 1.5**	**0.033**
Weeks per Year	28.2 ± 16.5	35.4 ± 15.2	0.085
Weeks Off per Year	10.4 ± 10.6	8.9 ± 11.4	0.596
*PROs at 1-Year Post-ACLR*			
Pedi-FABS	**20.6 ± 7.8**	**25.2 ± 4.7**	**0.012**
Pedi-IKDC	**88.0 ± 15.0**	**94.5 ± 7.9**	**0.049**
ACL-RSI	75.5 ± 23.1	76.5 ± 16.0	0.846
ACSI	**59.3 ± 12.4**	**66.0 ± 9.8**	**0.028**
Coachability	10.3 ± 1.9	10.9 ± 1.4	0.212
Concentration	**8.3 ± 2.8**	**9.7 ± 1.9**	**0.043**
Confidence and Achievement Motivation	**8.6 ± 2.0**	**10.0 ± 1.9**	**0.007**
Coping	8.2 ± 2.6	8.9 ± 2.2	0.320
Freedom from Worry	8.5 ± 2.3	8.2 ± 2.7	0.713
Goal Setting and Mental Preparation	**6.9 ± 2.9**	**8.8 ± 2.6**	**0.011**
Peaking under Pressure	**8.1 ± 2.7**	**9.5 ± 2.3**	**0.038**

*Significant differences between AIMS groups notated in bold. Groups split by high (AIMS_H_) or low (AIMS_L_) in total 1-year AIMS. AIMS: Athletic Identity Measurement Scale. ACLR: Anterior cruciate ligament reconstruction. SD: Standard deviation. Pedi-FABS: Hospital for Special Surgery Pediatric Functional Activity Brief Scale. Pedi-IKDC: Pediatric International Knee Documentation Committee Subjective Form. ACL-RSI: Anterior Cruciate Ligament-Return to Sport after Injury scale. ACSI-28: Athletic Coping Skills Inventory-28.

## 4 Discussion

### 4.1 Athletic identity during rehabilitation

This study represents an important step toward considering athletic identity as it relates to demographics, sport participation, and coping ability measures in youth athletes over the course of 1 year of rehabilitation following ACLR. The goals of this study were to determine how athletic identity, sport participation, and psychosocial variables change over 1 year, which variables relate to high/low athletic identity, and how these variables relate to a change in athletic identity over rehabilitation. The main findings include all AIMS scores decreasing during rehabilitation though only one domain and the total score were significant. Additionally, all PROs increased during rehabilitation other than ACSI-Confidence and Achievement Motivation. Finally, those with a higher baseline AIMS score had higher scores in physical and psychological PROs. The results indicate that most athletes returned to a similar level of activity as pre-injury and underwent significant improvement in knee function during rehabilitation.

Similar studies in mixed cohorts of youth and young adults reported AIMS scores between 44.2 and 57.5, which further support the current data (Manuel et al., 2002; Brewer et al., 2003; McKay et al., 2013; Padaki et al., 2018; McGinley et al., 2022). The observed decrease in AIMS confirms Brewer and Cornelius’ (2010) findings in adults who reported a gradual decline in athletic identity over 24 months post-ACLR, with the most substantial decline at 6–12 months post-ACLR. Moreover, the fact that all individual AIMS items decreased has multiple interpretations. First, athletes chose to pursue other aspects of their identity with more voracity during injury. The athlete role may become less salient in an effort to prevent potential disappointment and/or impact on self-esteem due to the inability to return to the pre-injury level of sport or to sport at all (Brewer and Cornelius, 2010). Additionally, instead of identity transition, it is possible that these athletes struggled to pursue new avenues (Podlog et al., 2011; Von Rosen et al., 2018) and found difficulty replacing that aspect of their identity. Regardless, results emphasize that a youth’s athletic identity is vulnerable in times of situational change such as injury, and an adequate support system is essential, as such (Newton et al., 2020).

In this cohort, only pre-ACLR AIMS scores were different between males and females. Certain studies report that sex and athletic identity are related (Babić et al., 2015; Şekeroğlu, 2017; McGinley et al., 2022) though the pattern is inconsistent. Others show no such relationships (Proios et al., 2012; Piatt et al., 2018; Costa et al., 2020; Rae and Jenkins, 2021), yet only McGinley et al. and Piatt et al. studied youth exclusively. As ACL injuries are three to six times more common in females (Arendt and Dick, 1995; Hewett et al., 2006; Bram et al., 2021), it may be especially important to consider athletic identity in addition to physical and other psychological aspects of ACL rehabilitation for female patients.

### 4.2 Sport participation

The results of this study suggest that training volume and activity level may be more significantly related to athletic identity than competition level, which may only loosely relate to training volume (Jayanthi et al., 2013). While studies have shown some correlations between athletic identity and sport participation factors—such as training volume, single- or multi-sport participation, and competition level—there is little consensus (Edison et al., 2021). Athletes who were part of the AIMS_H_ group scored consistently higher in all categories of training volume while also taking fewer weeks off though only the number of training days per week was significantly greater than the AIMS_L_ group. This finding correlates with the literature, which continues to suggest that training volume/intensity and athletic identity are likely related, though the specific measure of training volume is inconsistent (Lamont-Mills and Christensen, 2006; Anderson et al., 2009; Tasiemski and Brewer, 2011; Ahmadabadi et al., 2014; Piatt et al., 2018; Quinaud et al., 2020; Christino et al., 2021; McGinley et al., 2022). Those in the AIMS_INC_ group showed similar patterns of higher training volume as in the AIMS_H_ group, though only training weeks per year were significant. These results postulate that training volume may have a bearing on the ability or desire to recover athletic identity after injury. It is possible that those who participate more frequently in training for their sport also find a larger portion of their social interaction tied to that training, and they are more motivated to recover both their physical function and social network. Additionally, a higher AIMS score was associated with higher activity levels. The literature is unclear as to whether a relationship between AIMS and activity level exists at pre-ACLR (Reifsteck et al., 2013; Ohji et al., 2021). However, these data suggest that, in mid-to-late ACLR rehabilitation, those with higher athletic identity during rehabilitation were also those participating in more activity.

### 4.3 Physical function and coping ability

The AIMS_H_ group and AIMS_INC_ group showed better physical function scores in the same pattern as activity level, which is supported by past studies (Langford et al., 2009; Reifsteck et al., 2013; Ohji et al., 2021). However, the literature is somewhat conflicting as Hsu et al. (2021) did not observe a relationship between physical function and athletic identity. According to our results, those able to recover their knee function the best were also those successful in recovering/increasing their athletic identity and vice versa. The exact direction of this relationship is unknown as it may be that increased function affords greater ability to recover identity among athletic peers. Conversely, greater athletic identity may indicate athletes are more determined for successful recovery. Psychological readiness by ACL-RSI also appears to improve with rehabilitation, as expected.

As youth athletes may exhibit depressive symptoms and emotional trauma following injury due to exclusion from sport and a faltering network of peer relationships (Podlog et al., 2011; Von Rosen et al., 2018; Park et al., 2023), it is helpful for providers to understand how youth athletes’ coping abilities associate with their athletic identity (Manuel et al., 2002; Chow et al., 2016; McKay et al., 2019; Edison et al., 2021; Nyland and Pyle, 2022). In this study, the AIMS_H_ group showed greater (though not necessarily significant) coping abilities in every category except “Freedom from Worry.” It appears that those with elevated athletic identity at 1 year may also be those with higher baseline coping abilities as measured by the ACSI. This result is supported by Christino et al. (2021) who also observed improved coping abilities in highly specialized athletes—associated with higher athletic identity in their study—in all domains except Freedom from Worry. As Peaking under Pressure was the only ACSI-28 item to be significant in both the high AIMS 1-year group and the increased AIMS group, it may be valuable to anticipate future changes in athletic identity, regardless of pre-ACLR level. Overall, it appears that athletes who are more effective and motivated to recover their athletic identity were more effective at coping, perhaps as a result of their coping abilities minimizing identity loss during early rehabilitation. As Chmielewski et al. (2011) reported that psychosocial factors may be modifiable after ACLR, this result is promising for providers wishing to include psychosocial considerations in rehabilitation criteria.

### 4.4 Limitations and conclusion

The limitations of the current study include the low sample size of certain demographics and sport participation variables including race/ethnicity and recreational athletes. A variety of age and skill levels were also included in the study sample as no comparison of AIMS across adolescence exists to the best of our knowledge. While large differences in maturity and other factors may exist among this cohort, the literature does not identify age distinctions which may provide cutoff values. Moreover, the division of the cohort would hinder our ability to report on a large pediatric sample reflective of the region with limited prior literature on athletic identity during rehabilitation. Additionally, training volume and activity level measures may be skewed by the varying levels of clearance afforded to patients at that point; however, as recovery duration showed no significant differences between high/low AIMS and increased/decreased AIMS score, it is unlikely to be significant. Finally, patients were asked to answer PROs in their current injured state at pre-ACLR; thus, no healthy baseline was available for comparison and results relied on self-reported measures. However, the use of 1-year questionnaires may serve as a closer indicator of baseline than pre-surgery questionnaires due to the additional stressors associated with surgery.

This study was able to explore athletic identity as it relates to demographics, sport participation, and psychosocial factors in a pediatric population during rehabilitation. While results indicate that youth decline in their athletic identity even 1 year into rehabilitation, those who display increases in athletic identity and overall greater levels at post-ACLR appear to be those with increased physical function, activity level, and coping abilities. Specifically, the Pedi-FABS and Pedi-IKDC were associated with high 1-year AIMS scores, and higher ACSI-Peaking under Pressure was related to both high 1-year AIMS score and an increase between pre-ACLR and 1 year. Future studies should continue to investigate measures related to athletic identity over the course of recovery, in an age-stratified cohort, to assist practitioners in rehabilitation and improve the percentage of youth athletes able to return to sport and/or recover an integral aspect of their self-identity.

## Data availability statement

The raw data supporting the conclusions of this article will be made available by the authors, without undue reservation.

## Ethics statement

The studies involving humans were approved by University of Texas Southwestern Institutional Review Board. The studies were conducted in accordance with the local legislation and institutional requirements. The ethics committee/institutional review board waived the requirement of written informed consent for participation from the participants or the participants' legal guardians/next of kin because of the retrospective nature of the study.

## Author contributions

JM: Conceptualization, Formal analysis, Investigation, Visualization, Writing – original draft. ES: Investigation, Writing – review & editing. EG: Investigation, Writing – review & editing. HW: Data curation, Writing – review & editing. CP: Investigation, Writing – review & editing. HE: Conceptualization, Investigation, Resources, Writing – review & editing. PW: Conceptualization, Investigation, Resources, Writing – review & editing. SU: Formal analysis, Investigation, Project administration, Supervision, Writing – review & editing.
